# Ultraviolet inflorescence cues enhance attractiveness of inflorescence odour to *Culex pipiens* mosquitoes

**DOI:** 10.1371/journal.pone.0217484

**Published:** 2019-06-04

**Authors:** Daniel A. H. Peach, Elton Ko, Adam J. Blake, Gerhard Gries

**Affiliations:** Department of Biological Sciences, Simon Fraser University, Burnaby, BC, Canada; INRA-UPMC, FRANCE

## Abstract

Inflorescence patterns of ultraviolet (UV) absorption and UV-reflection are attractive to many insect pollinators. To understand whether UV inflorescence cues affect the attraction of nectar-foraging mosquitoes, we worked with the common house mosquito, *Culex pipiens* and with two plant species exhibiting floral UV cues: the tansy, *Tanacetum vulgare*, and the common hawkweed *Hieraciumm lachenalii*. Electroretinograms revealed that *Cx*. *pipiens* eyes can sense UV wavelengths, with peak sensitivity at 335 nm. Behavioural bioassays divulged that UV inflorescence cues enhance the attractiveness of inflorescence odour. In the presence of natural floral odour, female *Cx*. *pipiens* were attracted to floral patterns of UV-absorption and UV-reflection but preferred uniformly UV-dark inflorescences. Moreover, *Cx*. *pipiens* females preferred UV-dark and black inflorescence models to UV-dark and yellow inflorescence models. With feathers and pelts of many avian and mammalian hosts also being UV-dark and dark-coloured, foraging *Cx*. *pipiens* females may respond to analogous visual cues when they seek nectar and vertebrate blood resources.

## Introduction

Plant sugar, mainly in form of floral nectar, is the essential basic food for adult mosquitoes [1] that can serve as pollinators to the many plants they visit [2–6]. Floral semiochemicals are believed to attract mosquitoes to inflorescences [1,7,8], whereas visual floral cues were thought [9], and recently shown [10–12], to play a contributing role. Field observations suggest that mosquitoes most often visit light-coloured flowers [13–15] but preferential visitation to these types of flowers has yet to be rigorously tested [9]. Exclusively visual cues of oxeye daisy inflorescences did not attract mosquitoes in laboratory experiments [16] but olfactory oxeye daisy cues alone or in combination with visual cues did [16]. Both the yellow fever mosquito, *Aedes aegypti* (L.), and the northern house mosquito, *Culex pipiens* L., were more strongly attracted to a combination of olfactory and visual inflorescence cues than to olfactory inflorescence cues alone [12], revealing a contributing role of visual cues in mosquito attraction to inflorescences.

The effect of visual cues on mosquito behaviour is evident in further studies. Southern house mosquitoes, *Cx*. *quinquefasciatus*, did learn to associate visual cues with palatable and non-palatable solutions of sucrose and sucrose-NaCl, respectively [10]. Mosquitoes also preferred dark-coloured over white-coloured artificial inflorescences associated with sucrose solutions [11]; however, the presence of human observers and their associated odours (CO_2_) in these experiments could have altered the preferential response of mosquitoes.

The many visual inflorescence cues that attract pollinators include inflorescence shape, colour, and colour patterns [17–19]. The circular “bullseye” colour pattern of many inflorescences or their UV ‘bullseye’–with petals having UV-absorbing bases and UV-reflective apices–attract pollinators and guide them to the inflorescence centre [17,20–23]. The evolutionary “display” of inflorescences seem to factor in the UV-sensitivity (300–400 nm) [24] of their insect pollinators [25,26]. Studying the sensory capabilities of mosquito photoreceptors will allow us to understand the type of visual cues and signals that mosquitoes can sense and exploit during foraging and mate location. Electroretinograms (ERGs) with compound eyes of *Ae*. *aegypti* revealed receptor sensitivity peaks in the UV and yellow-green wavelength ranges [27], implying, e.g., that UV nectar guides of inflorescences could be exploited by UV-sensitive, nectar-foraging mosquitoes. Expectedly then, UV opsins were found in *Ae*. *aegypti* and *Anopheles gambiae* [28,29].

The UV-sensitivity of mosquitoes is also exploited in mosquito trapping programs that deploy both UV-light and CO_2_ as trap baits [30,31]. Other mosquito traps exhibit visual cues that emphasize contrast [32,33] which matters to host-foraging mosquitoes [34,35].

**Fig 1:**
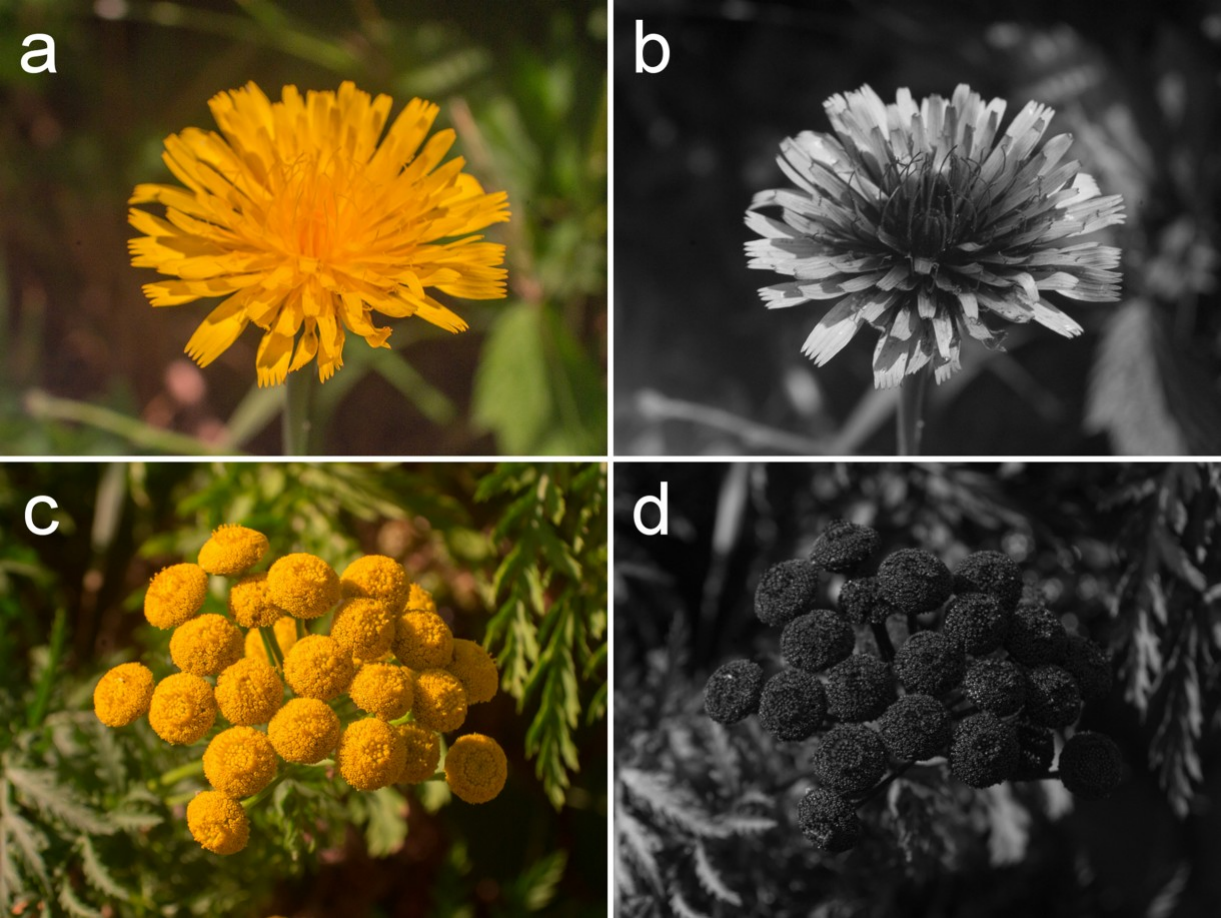
Photographs of common hawkweed and common tansy in the human-visible light range and UV light range. Inflorescences of common hawkweed, *Hieracium lachenalii* (**a,b**), and common tansy, *Tanacetum vulgare* (**c,d**), photographed with a custom-built camera capable of taking images in the human-visible light range (**a,c**) and UV light range (**b,d**). *Hieracium lachenalii* (**b**) displays a prominent UV “bullseye” with UV-absorbing petal bases and UV-reflective petal apices.

*Culex pipiens* is a nocturnal mosquito native to temperate Eurasia and established throughout temperate North America [36]. It vectors West Nile virus (WNV) [37] and avian malaria [38]. *Cx*. *pipens* visits many flowers of the Asteraceae [6,39,40], including the common tansy, *Tanacetum vulgare* [6]. To determine whether floral UV reflection and absorption patterns have a functional role in the context of nectar-foraging by mosquitoes, we used the common tansy, *Tanacetum vulgare*, which is UV-absorbing (Fig 1) and pollinated by *Cx*. *pipiens* [6], and the common hawkweed, *Hieracium lachenalii*, which exhibits a prominent UV bullseye (Fig 1) and is closely related to the king-devil hawkweed, *Hieracium pratense*, which is visited by several *Aedes* spp. [15].

Our objectives were (1) to determine both the ability of *Cx*. *pipiens* compound eyes to sense UV light, (2) to bioassay the effect of visual inflorescence cues (in the presence of inflorescence odour) on attraction of *Cx*. *pipiens*, (3) to study the effect of UV absorption and reflection patterns in *H*. *lachenalii* inflorescences on attraction of *Cx*. *pipiens*; and (4) to determine the specific characteristics of floral UV light cues, and possible interactions with floral colour cues, that mediate attraction of *Cx*. *pipiens*.

## Materials and methods

### Ethics approval

Ethics approval was not required for DP blood-feeding mosquitoes on his arms (Simon Fraser University Office of Research Ethics, personal communication). Plants were collected from the Burnaby campus of SFU, British Columbia, Canada between June-November 2017 and 2018, not requiring a collection permit for these plants which are neither endangered or protected.

### Experimental insects

We obtained mosquitoes from a laboratory colony of *Cx*. *pipiens* maintained at Simon Fraser University (SFU) in Burnaby, British Columbia, Canada. We sustained adult *Cx*. *pipiens* on a 10% sucrose solution, provided *ad libitum*, in mesh cages (30 × 30 × 46 cm high) maintained at 23–26 ^o^C, 40–60% RH, and a photoperiod of 14L:10D. Once a week, DP blood-fed adult females on his arm. For oviposition, we gave gravid females access to water in a circular glass dish (10 cm diameter × 5 cm high). We transferred egg rafts to water-filled trays (45 × 25 × 7 cm high) and provided larvae with NutriFin Basix tropical fish food (Rolf C. Hagen Inc., Montreal, QC, Canada). We transferred pupae with a 7-ml plastic pipette (VWR International, PA, USA) to water-filled 354-ml Solo cups covered with a mesh lid (Solo Cup Company, IL, USA). We released eclosed adults into mesh cages (30 × 30 × 46 cm high), transferred virgin females via aspirator to separate water-containing Solo cups, and provisioned them with a cotton ball soaked in a 10-% sucrose solution.

### Experimental plants

We collected inflorescences of *T*. *vulgare* and *H*. *lachenalii* from the Burnaby campus of SFU, British Columbia, Canada between June-November 2017 and 2018, not requiring a collection permit for these plants which are neither endangered or protected. We used inflorescences in experiments within four hours of collection.

### Electroretinograms

We determined the sensitivity of *Cx*. *pipiens* compound eyes to wavelengths in the UV and human-visible range (300–650 nm) using electroretinogram (ERG) recordings. We first cold-anesthetized, and then immobilized, each of fifteen 3- to 4-day-old *Cx*. *pipiens* females, ventral side up, on a piece of sticky tack (The Michaels Companies, Inc., TX, USA). We affixed this preparation to a glass microscope slide and placed it on a platform below a microscope (Wild M10, Leica Microsystems, ON, Canada). We used Leitz micromanipulators M (Leitz, Vienna, Austria) to insert glass microelectrodes into the left eye and the thorax of the immobilized female mosquito. Electrodes were formed with a micropipette puller (Model P-1000, Sutter Instrument Co., CA, USA), filled with a Ringer solution [41], fitted with a silver wire, and had a resistance of 1–10 MΩ.

We adapted the mosquito eyes to darkness, green light or UV light for 45 min prior to ERGs. The adapting lights consisted of a green- and a UV light-emitting diode (LED; B5B-433-B25, UV RLT350-0.3–15; Roithner LaserTechnik, Vienna, Austria), with nominal peak wavelengths of 525 nm and 351 nm, respectively (S1A Fig). We attached each LED to the terminal end of the fibre optic cable delivering stimulus light and positioned it such that the LED light shone on the same portion of the mosquito eye as the fiber optic cable. We performed each adaptation on five separately prepared mosquitoes, for a total of 15 mosquitoes.

We generated light stimuli using a 35-watt Xenon Arc light source (HPX-2000, Ocean Optics, Dunedin, FL, USA) and a fibre optic scanning monochromator (MonoScan 2000, Mikropak GmbH, Ostfildern, Germany). From this monochromator, light was transmitted through a 600-μm optical fibre (QP600-1-SR-B X, Ocean Optics, FL 32792, USA) fitted with a collimator (LC-4U-THD, Multimode Fiber Optics, Hackettstown, NJ, USA) and through a 0–2 stop circular variable neutral density wheel (fused silica (200–2500 nm); Reynard Corp., San Clemente, CA, USA) directly in front of a 20:80 beam splitter (“polka dot” 4–2001; Optometrics, Ayer, MA, USA). We transmitted 20% of the light to a calibrated cosine-corrector-fitted (CC-3-UV-S, Ocean Optics, Dunedin, FL, USA) spectrophotometer (HR-4000, Ocean Optics, Dunedin, FL, USA) to monitor and adjust the absolute irradiance of test stimuli. The remaining 80% of the transmitted light reached the eye of the test specimen via a cosine-corrector-fitted 1000-μm single fibre optic cable (PCU-1000-2-SS, Multimode Fiber Optics, Hackettstown, NJ, USA) with a Sub-Miniature-A (SMA) terminus. We opened a custom-built programmable shutter (R. Holland, Science Technical Centre, Simon Fraser University, Burnaby, BC, Canada) located between the beam splitter and the cosine corrector for 0.5 s every 10 s to expose the eye to a test stimulus at an intensity of 1.0 × 10^13^ photons/cm^2^/s and wavelength between 300–650 nm with a 5-nm bandwidth. We calibrated the response amplitudes to test stimuli against an intensity–response function to determine the sensitivity of the *Cx*. *pipiens* compound eye to those wavelengths. We amplified (Syntech Auto Spike, Syntech Inc., Hilversum, The Netherlands) electric potentials from the eye 100× in response to stimuli and recorded them with an electroantennogram (EAG) oscilloscope program (Syntech). We normalized the spectral sensitivities from individual compound eyes by the 97.5% quantile value of their sensitivity, and again normalized the mean spectral sensitivities for dark-, green-, and UV-adapted compound eyes in this fashion.

### Behavioural experiments

#### General design

**Fig 2:**
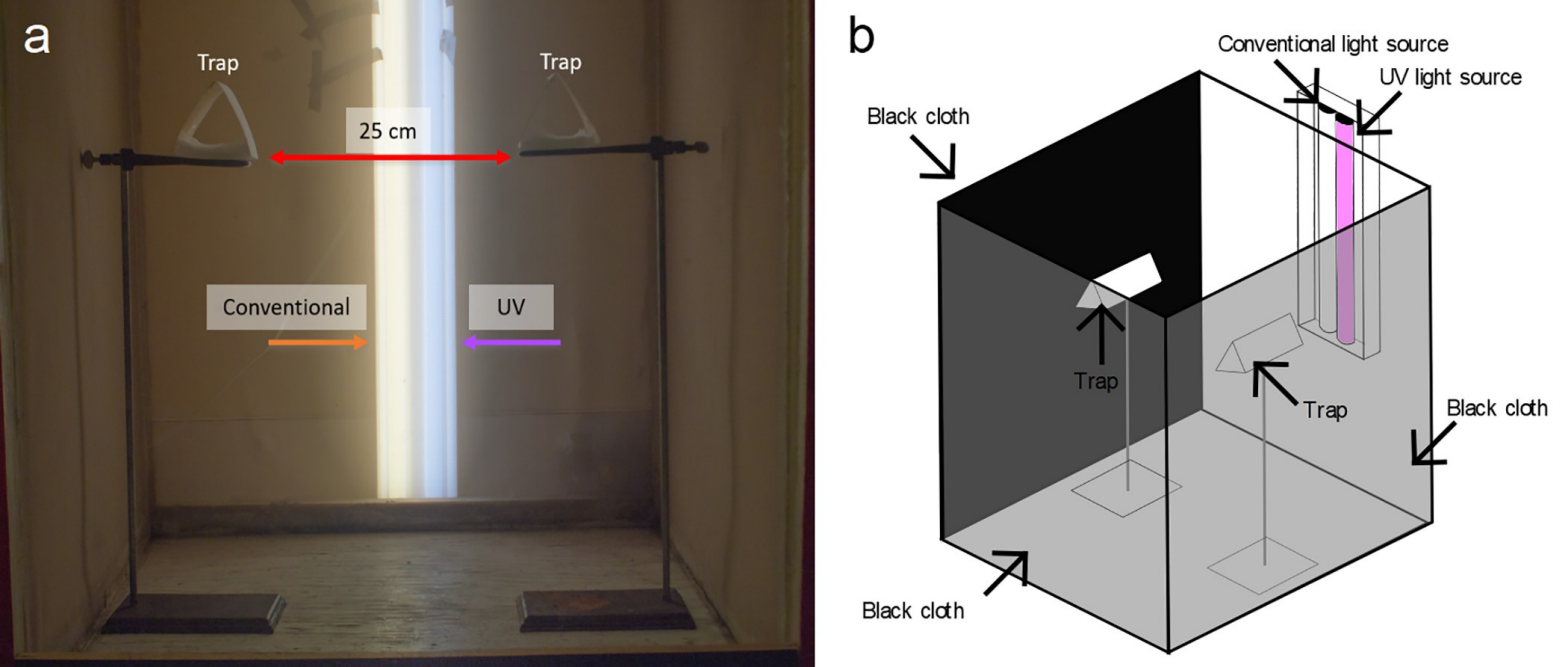
General design for behavioural bioassays. **(a)** Photo (front view) of the behavioural bioassay design, showing paired Delta traps inside a mesh cage, and the position of a conventional and UV light source. **(b)** Schematic drawing (top/lateral view) of the behavioural bioassay design, showing three side walls covered in black cloth, paired Delta traps, and the two light sources. For each bioassay replicate, 50 virgin, 1- to 3-day-old females were released into the cage, and trap captures were recorded 24 h later.

We ran experimental replicates in a windowless room without natural light at 23–26 ^o^C, a 40–60% relative humidity, and a photoperiod of 14L:10D. For each replicate, we released 50 virgin, 1- to 3-day-old females starved at least 24 h into a mesh cage (77 × 78 × 104 cm high), the front and lateral sides of which were covered with black cloth to minimize stray light entry, and the top and back were left uncovered (Fig 2). The cage center housed two burette stands separated by 25 cm, each stand carrying a Delta trap 50 cm above the cage floor (S2 Fig). We made traps from white or black cardstock (71.28 × 55.88 cm) (Staples Inc., MA, USA; ACCO Brands Corp., IL, USA) that we cut to size (15 × 30 cm), coated with adhesive (The Tanglefoot Company, MI, USA) on the inside, and then folded into a Delta-type trap (15 × 9 × 8 cm high). We terminated experiments after 24 h, at which time we scored trap captures and removed remaining mosquitoes from cages.

We illuminated cages with a shop light housing (Lithonia Lighting, GA, USA) placed vertically behind each cage and fitted with both a 1.22-m 10.0 UVB fluorescent tube (Zoo Med, San Luis Obispo, CA, USA) and a conventional 1.22-m fluorescent tube (F32T8/Tl835 Plus, Phillips, Amsterdam, Netherlands) (Fig 2B and 2C). We did not control for intensity of the conventional or UV lights. We connected the housing to a timer set to the same photoperiod (14L:10D) as the room lights.

#### Effect of visual inflorescence cues under UV light on *Cx*. *pipiens* attraction (Exps 1 and 2)

**Fig 3:**
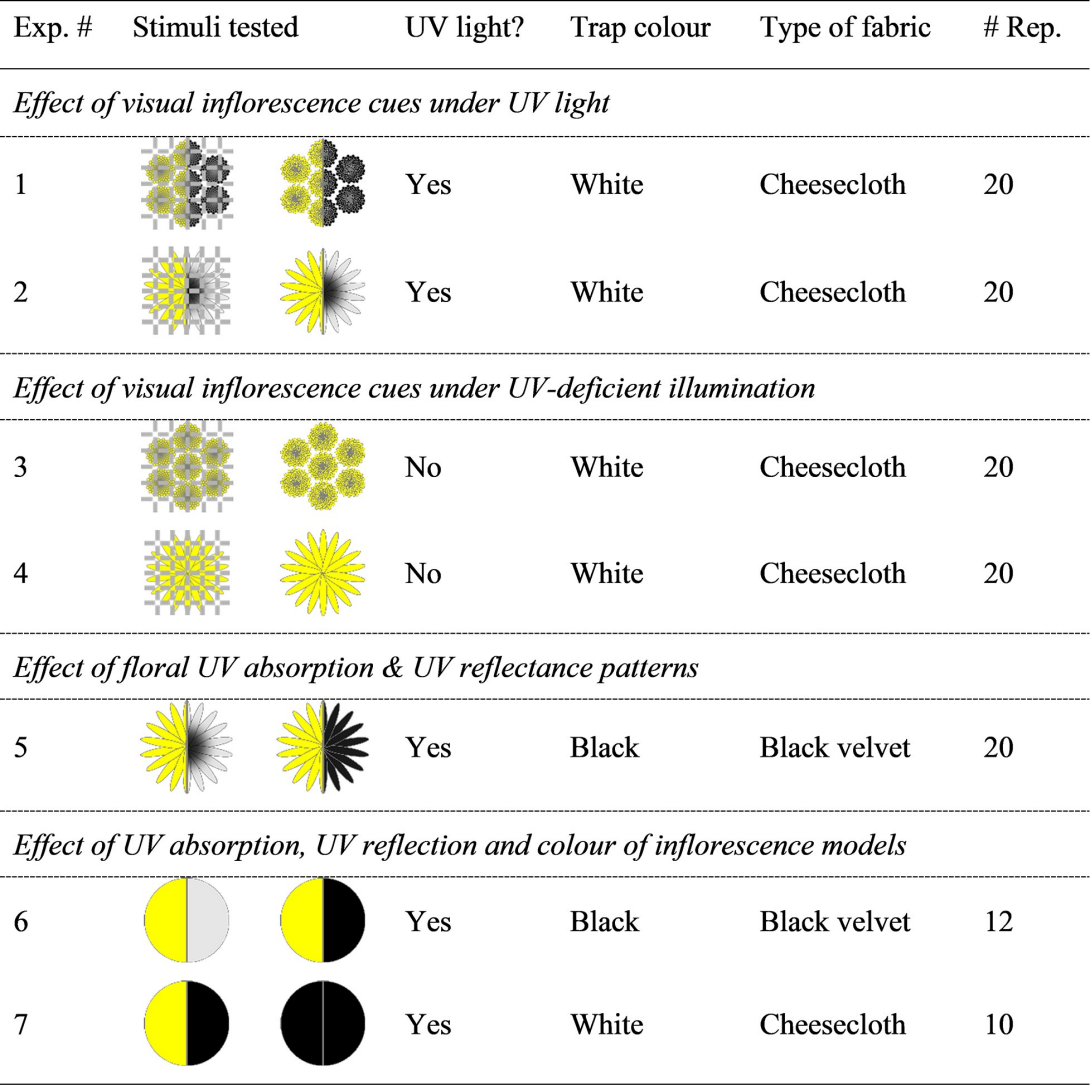
Summary of the experimental design to test attraction of female *Culex pipiens* to inflorescences of *Hieracium lachenalii* and *Tanacetum vulgare*, or to inflorescence models. Test stimuli are presented in schematic drawings, with left and right sections presenting the human-visible and UV light image, respectively; grey and black in the UV light image indicate UV reflection (UV-bright) and UV absorption (UV-dark), respectively; hatched lines indicate that the inflorescence was covered by cheese cloth; odour from natural inflorescences was present in all experiments (see methods for details).

In experiment 1 (Fig 3), treatment and control stimuli consisted of a freshly cut *T*. *vulgare* inflorescence with its stem inserted into a water-filled vial (4-ml) through a pre-punctured hole in Parafilm (Bemis Company Inc., WI, USA) that covered the vial opening. We placed each vial horizontally into a trap such that the inflorescence faced the light housing fitted with both a UV and a conventional fluorescent tube (see above). To determine the (additive) effect of visual cues on the attractiveness of *T*. *vulgare* inflorescences, we placed the vial containing the treatment inflorescence on top of cheesecloth (Cheesecloth Wipes, VWR International, PA, USA) and occluded the vial containing the control inflorescence with cheesecloth. Experiment 2 (Fig 3) was identical in design except that we tested *H*. *lachenalii* instead of *T*. *vulgare* inflorescences.

#### Effect of visual inflorescence cues under UV-deficient illumination on *Cx*. *pipiens* attraction (Exps 3 and 4)

The design of experiment 3 (Fig 3) was identical to that of experiment 1 except that we placed a sheet of polycarbonate (30.48 × 91.44 × 0.3175 cm thick; Lexan, SABIC, Riyadh, Saudi Arabia) with minimal UV transmission (S1 Fig) in front of the UV light source. This design essentially eliminated the visibility of the bullseye pattern from the inflorescence. Experiment 4 (Fig 3) was identical in design except that we tested *H*. *lachenalii* instead of *T*. *vulgare* inflorescences.

#### Effect of floral UV absorption & reflectance patterns on *Cx*. *pipiens* attraction (Exp 5)

The design of experiment 5 (Fig 3) was identical to that of experiment 4 except that we (1) placed each vial with its inflorescence on top of black velvet (Suzhou Joytex International Co. Ltd, Jiangsu, China), (2) deployed black instead of white delta traps, and (3) treated inflorescences to alter their bullseye (the characteristic UV absorption and reflection pattern). We treated the upper surface of petals of treatment inflorescences with a “sunscreen mix” of UV-absorbing Parsol 1789 and Parsol MCX (50:50 w/w; Sigma-Aldrich, ON, Canada) formulated in canola oil [adapted from 22]), and the upper surface of petals of control inflorescences with canola oil only. In addition, we treated the receptacle of control inflorescences with the “sunscreen mix” to ensure “odour symmetry” between treatment and control inflorescences.

#### Effect of UV light absorption, UV reflectance and colour of inflorescence model on attraction of mosquitoes (Exps 6 and 7)

In experiment 6 (Fig 3), we compared the attractiveness of yellow model flower discs (2.5 cm diameter) that exhibited either a uniformly UV-dark or a uniformly UV-bright appearance. We prepared the discs from yellow printer paper (International Paper, TN, USA), and painted treatment discs with clear nail polish (Coty Inc., NY, USA) rendering them dark in the UV range while maintaining their yellow, human-visible colouration. Using an inkjet printer, we printed control discs with a yellow ink that maintained their UV reflectance but rendered them darker to mimic the darkened appearance of nail polish-painted treatment discs. To ensure “odour symmetry” of the treatment and the control disc, we paired the nail polish- and inkjet- treated disks using their untreated side for contact. We then placed each disc pair into a black trap containing a *H*. *lachenalii* inflorescence which we occluded with black velvet to provide olfactory but not visual cues. In treatment and control traps, the nail polish-painted side and the yellow ink-printed side, respectively, of the paired discs leaned against the occluded inflorescence at a 45^o^ angle relative to the trap bottom and faced the light housing.

In experiment 7 (Fig 3), we explored a potential additive effect of floral colour (yellow) on the combined effect of floral odour and UV darkness on mosquito attraction. We modified the design of experiment 6 in that we prepared model flower control discs from black cardstock and model flower treatment discs from yellow printer paper, painting both discs with clear nail polish which renders them UV-dark. We also replaced black traps with white traps, and black velvet with cheesecloth.

### Spectral analyses

We measured the spectral reflectance of white cardstock, cheesecloth, black cardstock, and black velvet (all S1D Fig), and of *T*. *vulgare* and *H*. *lachenalii* inflorescences (S1E Fig) with a JAZ spectrometer (Ocean Optics Inc., Dunedin, FL, USA). Measurements covered a range of 300–700 nm and were corrected to absolute diffuse reflectance by a 99% Spectralon reflectance standard (SRS-99-010, Labsphere, NH, USA). We also took spectral reflectance measurements from *H*. *lachenalii* inflorescences (center and perimeter) that were (*i*) coated with canola oil (100% Pure Canola Oil, Richardson International, MB, Canada) (S1F and S1G Fig), or (*ii*) coated with a sunscreen mixture (50:50 w/w Parsol 1789 and Parsol MCX, Sigma-Aldrich, ON, Canada) formulated in canola oil (60:40 w/w sunscreen mixture) (S1B and S1C Fig). Furthermore, we took spectral reflectance measurements of yellow UV-bright disks, yellow UV-dark disks, and black UV-dark disks (S1H Fig).

We measured the absolute irradiance of 48-inch fluorescent UV bulbs (Zoo Med) and conventional bulbs (Philips, Amsterdam, Netherlands) deployed in bioassays, with or without a Lexan Polycarbonate filter that blocked UV transmissions (S1B and S1C Fig), with a calibrated spectrophotometer (HR-4000, Ocean Optics) using SpectraSuite software (Ocean Optics). We collected light using a cosine corrector (CC-3-UV-S, Ocean Optics) placed in the center of the cage (77 × 78 × 104 cm high) at a height of 50 cm.

### UV photography

We took UV photographs of *T*. *vulgare* and *H*. *lachenalii* inflorescences using a custom lens mounted to an Olympus E-PM1 camera (Olympus, Tokyo, Japan) modified for spectral sensitivity covering both the UV (< 400 nm) and human-visible light range (400–700 nm) (Dr. Klaus Schmitt, Weinheim, Germany, uvir.eu). We used an UV/IR filter (Baader Plantarium, Mammendorf, Germany) and a U-filter (Baader Plantarium, Mammendorf, Germany) for human-visible and UV images, respectively.

### Statistical analyses

We analyzed behavioural data using SAS statistical software version 9.4 (SAS Institute Inc., Cary, NC 27513, USA), excluding from analyses experimental replicates with no mosquitoes responding. We compared mean proportions of responders to paired test stimuli using a binary logistic regression model and worked with back-transformed data to obtain means and confidence intervals.

## Results

### Electroretinograms (ERGs)

**Fig 4:**
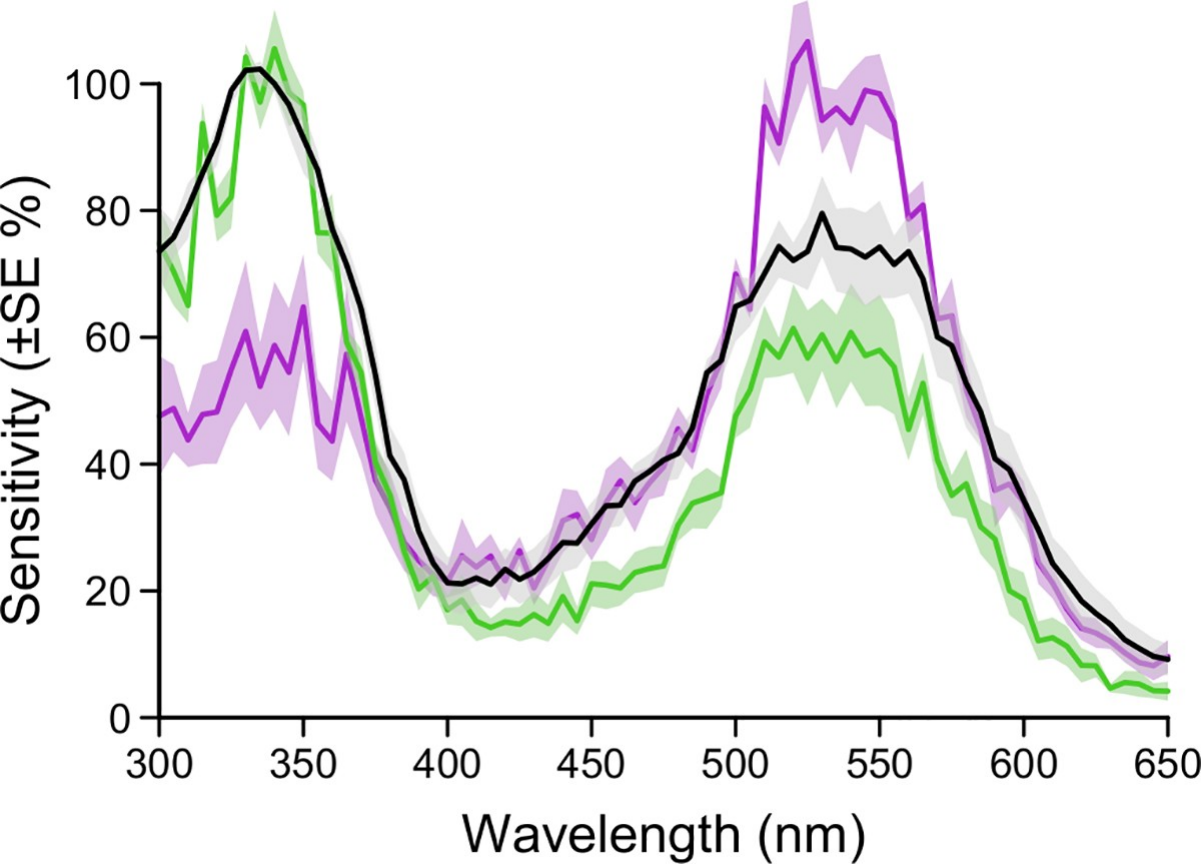
Spectral sensitivity of *Culex pipiens* compound eyes. Electroretinograms (ERGs) showing the mean spectral sensitivity of compound eyes of 3- to 4-day-old female *Culex pipiens* that were dark-adapted (black lines; n = 5), green-adapted (green lines; n = 5), or UV-adapted (purple lines, n = 5). The shaded area around each line represents the standard error of the spectral mean.

In ERG recordings following dark adaptation, *Cx*. *pipiens* eyes (n = 5) exhibited a spectral sensitivity peak in the UV range (335 nm) and the green range (540 nm) (Fig 4). Adaptations of eyes to green light (n = 5) or UV light (n = 5) induced sensitivity changes to green or UV light (Fig 4). As expected, UV-adapted eyes became less sensitive to UV light (300–400 nm), whereas green-adapted eyes became less sensitive in the visual range (400–650 nm).

### Spectral analyses

In the human-visible range, the inflorescence of both species appears yellow, strongly reflecting light above 500 nm.

Apices of *H*. *lachenalii* petals exhibited peak UV reflectance around 360 nm (S1 Fig; Infl. perimeter, untreated). Treatment of inflorescences with the canola oil/sunscreen mix lowered their UV light reflectance to < 5% (S1 Fig; Infl. perimeter, oil/sunscreen treated). Treatment of inflorescences with the canola oil control also lowered their UV light reflectance (S1 Fig; Infl. perimeter, oil treated) but not to a level below the natural variance recorded from other *H*. *lachenalii* inflorescences.

The composite inflorescences of *T*. *vulgare* exhibited negligible (<5%) UV reflectance below 400 nm (S1E Fig). The central and distal portions of *T*. *vulgare* inflorescences did not differ in reflectance spectra. In the human-visible range, peak reflectance occurred around 700 nm.

### Behavioural experiments

#### Effect of visual inflorescence cues under UV light on *Cx*. *pipiens* attraction (Exps 1 and 2)

**Fig 5:**
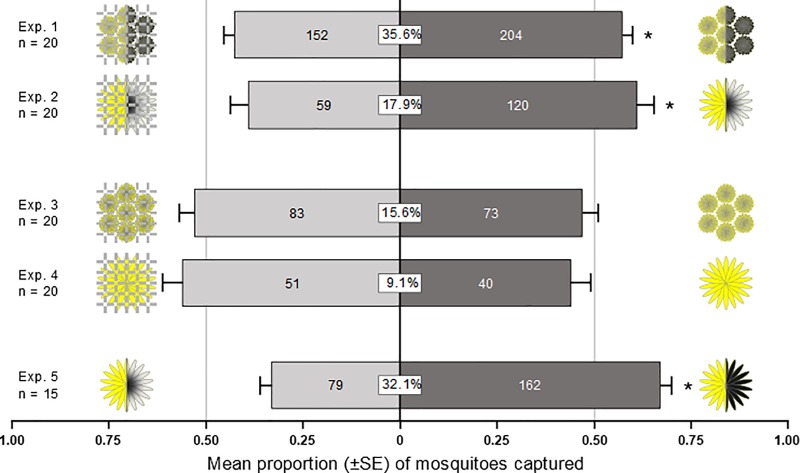
Effect of visual and olfactory inflorescence cues on trap captures of 1- to 3-day-old female *Culex pipiens*. Inflorescences of *Hieracium lachenalii* (Exp. 1) and *Tanacetum vulgare* (Exp. 2), respectively, are shown in schematic drawings, with left and right sections presenting the human-visible and UV-light image, respectively; hatched lines indicate the inflorescence was covered by cheese cloth. Visual inflorescence cues did enhance the effect of inflorescence odour under UV light (Exps. 1, 2) but did not under UV-deficient illumination (Exps. 3, 4). Uniformly UV-dark *H*. *lachenalii* inflorescences (as a result of sunscreen treatment) were more attractive than inflorescences with the natural UV absorption and UV reflectance pattern (Exp. 5). Numbers in bars indicate total number of mosquitoes responding. The boxed number in the centre of bars shows the response ratio (total number of mosquitoes captured divided by the total number of mosquitoes released expressed as percentage). For each experiment, an asterisk indicates a significant preference for a test stimulus (binary logistic regression model; p < 0.05).

When given a choice of either olfactory inflorescence cues alone (inflorescence under cheese cloth) or both olfactory and visual inflorescence cues (inflorescence on top of cheese cloth), female *Cx*. *pipiens* significantly preferred the bimodal *T*. *vulgare* inflorescence cue complex (z = 2.75, p = 0.006; Fig 5, Exp. 1) and the bimodal *H*. *lachenalii* inflorescence cue complex (z = 4.44, p < 0.0001; Fig 5, Exp. 2).

#### Effect of visual inflorescence cues under UV-deficient illumination on *Cx*. *pipiens* attraction (Exps 3 and 4)

When we presented *Cx*. *pipiens* females with the same choices as in preceding experiments 1 and 2 but under UV light-deficient illumination, these females no longer showed a preference for the bimodal (olfactory, human-visible) inflorescence cue complex of *T*. *vulgare* (z = -0.8, p = 0.42; Fig 5, Exp 3) or of *H*. *lachenalii* (z = -1.14, p = 0.26; Fig 5, Exp 4).

#### Effect of inflorescence UV reflectance and absorbance pattern on *Cx*. *pipiens* attraction (Exp 5)

Given a choice of (uncovered) inflorescences that were either uniformly UV-dark (treated with canola oil/sunscreen mix) or that still exhibited the UV bullseye (treated with canola oil control), female *Cx*. *pipiens* significantly preferred the former treatment (z = 5.21, p <0.0001; Fig 5, Exp 5).

#### Effect of UV absorption, UV reflection and colour of inflorescence models on *Cx*. *pipiens* attraction (Exps 6 and 7)

**Fig 6:**
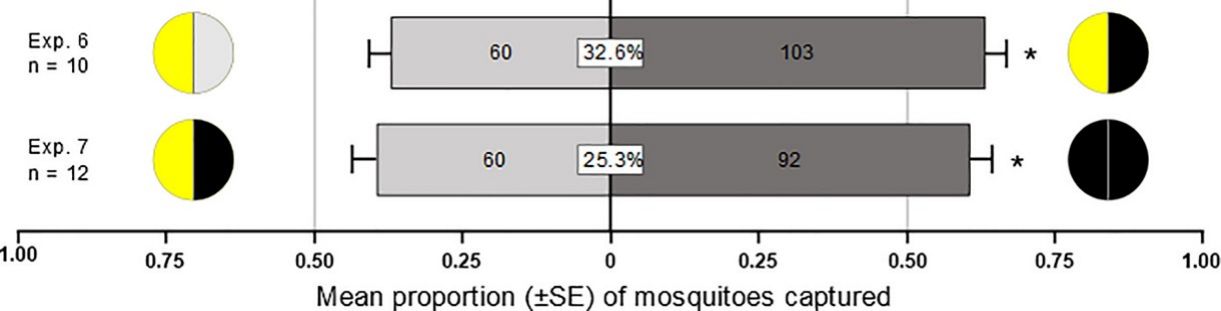
Effect of UV absorption, UV reflection and colour of inflorescence models in the presence of inflorescence odour (occluded inflorescence) on trap captures of 1- to 3-day-old female *Culex pipiens*. Inflorescence models are shown in schematic drawings, with left and right sections presenting the human-visible and UV light image, respectively. Yellow UV-dark models were more attractive than yellow UV-bright models (Exp. 6), whereas black UV-dark models were more attractive than yellow UV-dark models (Exp. 7), indicating an interaction between UV-darkness and colour. Numbers in bars indicate the total number of mosquitoes responding. The boxed number in the centre of bars shows the response ratio (total number of mosquitoes captured divided by the total number of mosquitoes released expressed as percentage). For each experiment, an asterisk indicates a significant preference for a test stimulus (binary logistic regression model; p < 0.05).

When we presented *Cx*. *pipiens* females (in the presence of *H*. *lachenalii* inflorescence odour) with a choice of yellow floral models which were either uniformly UV-bright or UV-dark, these females selected significantly more often the UV-dark model (z = 3.31, p = 0.0009; Fig 6, Exp 6).

When we presented *Cx*. *pipiens* females (in the presence of *H*. *lachenalii* inflorescence odour) with a choice of UV-dark floral models that were either yellow or black (in the human-visible range), these females selected significantly more often the black model (z = 2.58, p = 0.01; Fig 6, Exp 7).

## Discussion

Our findings indicate that (1) compound eyes of *Cx*. *pipiens* can sense UV light; (2) visual inflorescence cues render inflorescence odour more attractive to *Cx*. *pipiens*; (3) the UV “bullseye” of *H*. *lachenalii* inflorescences (Fig 1B) attracts *Cx*. *pipiens*; (4) the UV-dark trait of inflorescences is a strong driver of *Cx*. *pipiens* attraction, and (5) stimuli dark in both human visible light and UV light are most attractive to *Cx*. *pipiens*. Below, we shall elaborate on these findings.

### Compound eyes of *Cx*. *pipiens* can sense UV light, possibly using their photoreceptors R7 and R8

To determine the heretofore unknown spectral sensitivity of *Cx*. *pipiens* compound eyes, we conducted electroretinogram recordings, exposing eyes to 5-nm bandwidth of light in the UV and human-visible light range (300–650 nm). The recordings revealed that UV light of 335-nm wavelength and green light of 540-nm wavelength elicit the strongest receptor potentials (voltages) from *Cx*. *pipiens* eyes (Fig 4). These results indicate the presence of at least one UV-sensitive photoreceptor in *Cx*. *pipiens* eyes.

The spectral sensitivity of *Cx*. *pipiens* resembles that of other dipterans [42,43], particularly that of the yellow fever mosquito, *Ae*. *aegypti*, which exhibits peak spectral sensitivity in the UV (323–345 nm) and green (523 nm) ranges [27]. Similar to most dipterans, each ommatidium in *Ae*. *aegypti* contains eight photoreceptor cells (R1-8) [44]. The six outer photoreceptors (R1-6) express a longwave-sensitive opsin (rhodopsin Aaop1) [44], whereas the two inner photoreceptors (R7,8) express longwave-, UV- or blue-sensitive opsins depending on the eye region [28,29,45]. Interestingly, there is structural similarity of ommatidia in *Ae*. *aegypti* and *Cx*. *pipiens* [46,47], and similar sets of longwave-, UV- and blue-sensitive opsins are present in *Ae*. *aegypti* and *Cx*. *quinquefasciatus* [48,49], a sister species of *Cx*. *pipiens* [50,51]. All these facts coupled with the results of our ERG recordings (Fig 4) support the inference that *Cx*. *pipiens* and *Ae*. *aegypti* have similar complements of photoreceptors and comparable opsin expressions.

Following exposure to either UV light or green light, *Cx*. *pipiens* eyes became less sensitive to UV light and to green light (Fig 4), respectively. If only a single photoreceptor type were to be responsible for theses adaptions, we would expect similar sensitivity changes following pre-exposure to either UV or green light. The observed dissimilar sensitivity changes following UV or green light pre-exposure (Fig 4) suggest that both a green-sensitive and a UV-sensitive photoreceptor contributed to the ERG responses. Assuming this interpretation is correct, our data would provide supporting evidence that the central photoreceptors (R7, R8) of *Cx*. *pipiens* ommatidia express either a green or a UV opsin, unlike photoreceptors R1-6, which all express an identical green or blue-green opsin in other Diptera [44,52]. Our light adaptation experiments revealed no evidence for a blue-sensitive receptor contributing to the response. We expected this because the blue opsin is likely expressed at low levels in the central region of *Cx*. *pipiens* eyes [28], but photoreceptor responses may have also been affected by the green adaptation light.

The spectral sensitivity of *Cx*. *pipiens* eyes in the UV range (Fig 4) can be attributed to (*i*) the response of a UV-sensitive opsin in the central photoreceptors (R7 or R8), (*ii*) a UV-sensitizing pigment in photoreceptors R1-R6, or (*iii*) both. If *Cx*. *pipiens* and *Ae*. *aegypti* were to show similar opsin expression, then photoreceptor R7 in the central eye region (where recordings were performed) would presumably express a UV opsin with a sensitivity peak of ~330 nm. Yet, the recorded sensitivity peak (335 nm; Fig 4) may also have also originated from photoreceptors R1-R6 that—due to their abundance and size—are the main contributors to electroretinogram responses of dipteran eyes [43,53]. A UV-sensitizing pigment has been found in photoreceptors R1-6 of the common vinegar fly, *Drosophila melanogaster* [42], in the tiger mosquito, *Aedes albopictus* [52], but not in *Ae*. *aegypti* [52]. Several brachyceran flies express 3-hydroxy-retinal as a UV-sensitizing pigment in their photoreceptors R1-6 [52]. Within the Nematocera, males of black flies (Simuliidae) express a different UV-sensitizing pigment (presumably retinol) in their photoreceptors R1-6, generating a separate sensitivity peak at 340 nm [52]. There is also preliminary evidence for a similar screening pigment in the Asian tiger mosquito, *Ae*. *albopictus* [G. Belušič pers. comm.;52].

### Visual inflorescence cues render inflorescence odour more attractive to *Cx*. *pipiens*

To ascertain that visual inflorescence cues contribute to the overall attractiveness of *H*. *lachenalii* and *T*. *vulgare* inflorescences, we isolated the effect of visual cues by testing inflorescences as a trap bait that were occluded, or not, with cheese cloth, presenting mosquitoes with a choice of either olfactory cues alone (inflorescence occluded) or both olfactory and visual cues (inflorescence not occluded). Significantly greater captures of *Cx*. *pipiens* females in traps baited with a non-occluded inflorescence (Fig 5, Exps 1 and 2) established a contributing effect of visual cues to the inflorescence attractiveness. These results are not surprising in light that diverse taxa of floral visitors exploit visual inflorescence cues [18,54–56], and that foraging mosquitoes respond to visual cues when they seek vertebrate hosts [9]. Our results also confirm previous findings that visual inflorescence cues are part of a multimodal cue complex that guides nectar-foraging mosquitoes to inflorescences [12]. Similarly, there is synergy between visual and olfactory inflorescence cues that guide nectar-foraging wild hawkmoths [55]. However, attraction of mosquitoes to visual inflorescence or visual vertebrate cues appears to be contingent upon the presence of other cues such as odourants or CO_2_ [35,57] that initiate the foraging behaviour.

### Patterns of UV absorption and UV reflection displayed in “the bullseye” of *H*. *lachenalii* inflorescences attract *Cx*. *pipiens*

To determine whether UV light contributes to the attractive effect of visual inflorescence cues, we either eliminated UV wavelengths from illuminating light sources or altered UV reflections from inflorescences. To produce UV-deficient illumination, we placed a Lexan filter in front of illumination devices, thereby effectively eliminating the UV bullseye from *H*. *lachenalii* inflorescences. Under UV-deficient light, female *Cx*. *pipiens* no longer showed a preference for inflorescences with bimodal (olfactory, human-visible) cues (Fig 5, Exps 3 and 4), suggesting that it is the bullseye contrast of UV-absorbed and UV-reflected light that–together with floral odourants–guide mosquitoes to inflorescences. However, uniformly UV-dark *H*. *lachenalii* inflorescences, following treatment with a canola oil/sunscreen mix (S1 Fig), were even more attractive to *Cx*. *pipiens* than control inflorescences that retained the bullseye contrast (Fig 5, Exp 5), indicating that *Cx*. *pipiens* females prefer UV-dark inflorescences. These findings are surprising in light of previous reports that the treatment of silverweed cinquefoil, *Argentina anserina*, inflorescences with a sunscreen mix (that disrupted the UV bullseye) decreased insect visitation and behaviour compared to control inflorescences which exhibited the usual UV bullseye phenotype [22]. A potential role of UV light on attraction of mosquitoes to visual inflorescence cues could not be detected in other studies because wavelengths only in the human-visible range were considered [10–12]. The possibility that UV-deficient illumination also alters mosquito behaviour has not yet been investigated.

### The UV-dark attribute of inflorescences is a strong driver of *Cx*. *pipiens* attraction and its appeal is enhanced by dark (black) colour

In choice experiments with uniformly UV-dark or UV-bright yellow or black inflorescence models (in the presence of natural inflorescence odour), *Cx*. *pipiens* females preferred UV-dark over UV-bright yellow models and black UV-dark over yellow UV-dark models (Fig 6), supporting the significance of floral UV reflectance as a visual foraging cue (see Exp. 5). Neither low UV contrast (Fig 1, Exps 1, 2) nor high UV contrast (Fig 6, Exp 6) between the test stimulus and the trap background seem to affect mosquito attraction. Other studies found mosquito attraction to dark-coloured objects or to objects with light and dark contrast [34,35]. Previous conclusions that diurnally-active dipteran pollinators prefer inflorescence patterns of UV-absorption and UV-reflection [22] may be attributed to the fact that pertinent experiments were performed on diurnally-active species rather than crepuscular-active nectar-foraging mosquitoes. Moreover, *Cx*. *pipiens* forage on many inflorescences (e.g., *Tanacetum vulgare*, *Achillea millefolium*, *Leucanthemum vulgare* [6,13,40]) that are uniformly UV-dark [58–60].

The preference of nectar-foraging *Cx*. *pipiens* for black UV-dark inflorescence models over yellow UV-dark models implies that attractive stimulus traits may be intensity- rather than spectrally-based, with mosquitos being attracted to models that reflect relatively little light across their entire visual range (300–600 nm). This phenomenon is reminiscent of host-foraging mosquitos that are attracted to dark objects, such as the UV-absorbing dark plumage and pelage of many avian and mammalian hosts [61–63]. It seems that nectar and host-foraging mosquitoes respond to analogous but contextually different visual resource cues [64].

If *Cx*. *pipiens* females were to exclusively use the R1-6 photoreceptors to inform orientation behaviour, this would bypass the colour vision circuits associated with the photoreceptors R7 and R8, and would possibly explain the preference for dark objects. The R1-6 photoreceptors are thought to provide an achromatic visual channel in flies [65] and have only a limited role in colour vision [66]. If, like other flies, *Cx*. *pipiens* were to possess a UV-sensitising pigment in the R1-6 photoreceptors, these photoreceptors would be expected to have a broadband sensitivity (300–600 nm) that would only be able to distinguish among objects based on brightness (intensity of perceived reflected light).

## Conclusion

We have shown that nectar-foraging *Cx*. *pipiens* females respond to both olfactory and visual inflorescence cues. UV-sensitive eyes enable *Cx*. *pipiens* females to detect, and discern between, floral patterns of UV-absorption and UV-reflection, with preference for inflorescences with low reflection of both human-visible and UV light. With feathers and pelts of many avian and mammalian hosts being similarly dark, foraging mosquitoes may respond to analogous but contextually different visual cues when they seek nectar and vertebrate blood resources.

## Supporting information

S1 Fig Irradiances and reflectance spectra of illuminating lights, traps, and test stimuli deployed in behavioral experiments 1–7. (**a**) Irradiance spectra of the green and UV LEDs used for electroretinogram recordings. (**b,c**) Combined irradiance spectra of a conventional (conv.) lamp and an ultraviolet (UV) fluorescent lamp with or without a polycarbonate (poly.) sheet that reduces UV transmission. (**d**) Diffuse reflectance spectra of materials used to custom-build Delta traps (white or black cardstock) or to occlude *Hieracium lachenalii* inflorescences (cheesecloth, black velvet). Note: white cardstock reflectance above 400 nm was measured through a polycarbonate sheet to eliminate the effect of optical brighteners which fluoresce under UV light. (**e**) Diffuse reflectance of *H*. *lachenalii* inflorescences (center and perimeter) and *Tanacetum vulgare*. (**f,g**) Diffuse reflectance spectra of *Hieracium lachenalii* inflorescences (center and perimeter) either not treated, treated with canola oil, or treated with a mix of canola oil and sunscreen. (**h**) Diffuse reflectance spectra of inflorescence models prepared from disks of yellow printer paper (yellow) or black cardstock (black) treated with either yellow inkjet printer ink or clear nail polish.
(TIF)

S2 Fig Ultraviolet (UV) and human visible photographs of Delta traps and stimuli (natural inflorescences or inflorescence models) tested in behavioral experiments 1–7. (TIF)

S1 Dataset Experimental data. (XLSX)
