# Supplementary figures and images for: Ultraviolet inflorescence cues enhance attractiveness of inflorescence odour to Culex pipiens mosquitoes
Source: PLoS One. 2019 Jun 4;14(6):e0217484. doi: 10.1371/journal.pone.0217484 (PMC6548384; doi:10.1371/journal.pone.0217484)

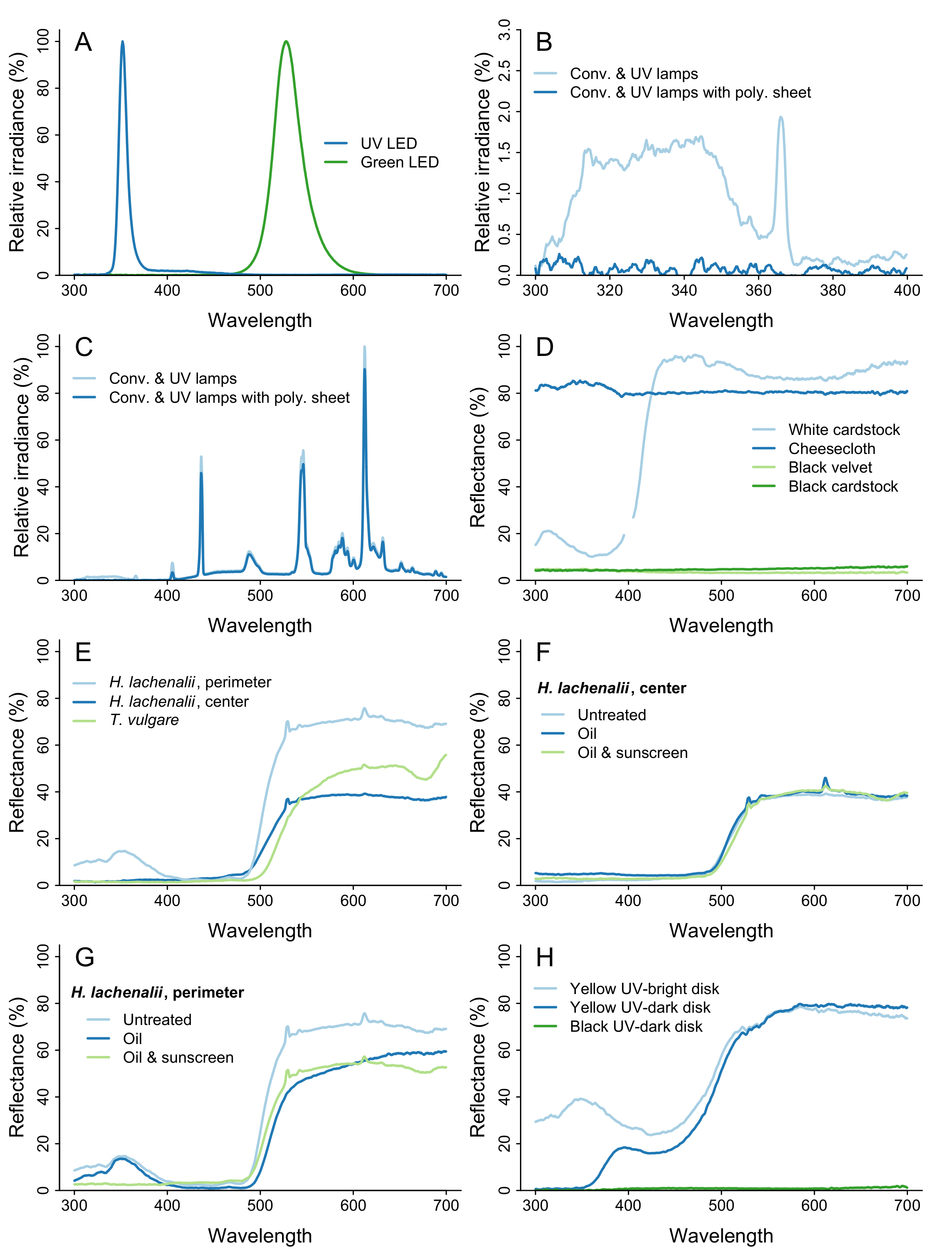

Supplement: S1 Fig — (a) Irradiance spectra of the green and UV LEDs used for electroretinogram recordings. (b,c) Combined irradiance spectra of a conventional (conv.) lamp and an ultraviolet (UV) fluorescent lamp with or without a polycarbonate (poly.) sheet that reduces UV transmission. (d) Diffuse reflectance spectra of materials used to custom-build Delta traps (white or black cardstock) or to occlude Hieracium lachenalii inflorescences (cheesecloth, black velvet). Note: white cardstock reflectance above 400 nm was measured through a polycarbonate sheet to eliminate the effect of optical brighteners which fluoresce under UV light. (e) Diffuse reflectance of H. lachenalii inflorescences (center and perimeter) and Tanacetum vulgare. (f,g) Diffuse reflectance spectra of Hieracium lachenalii inflorescences (center and perimeter) either not treated, treated with canola oil, or treated with a mix of canola oil and sunscreen. (h) Diffuse reflectance spectra of inflorescence models prepared from disks of yellow printer paper (yellow) or black cardstock (black) treated with either yellow inkjet printer ink or clear nail polish. (TIF) [file pone.0217484.s001.tif]

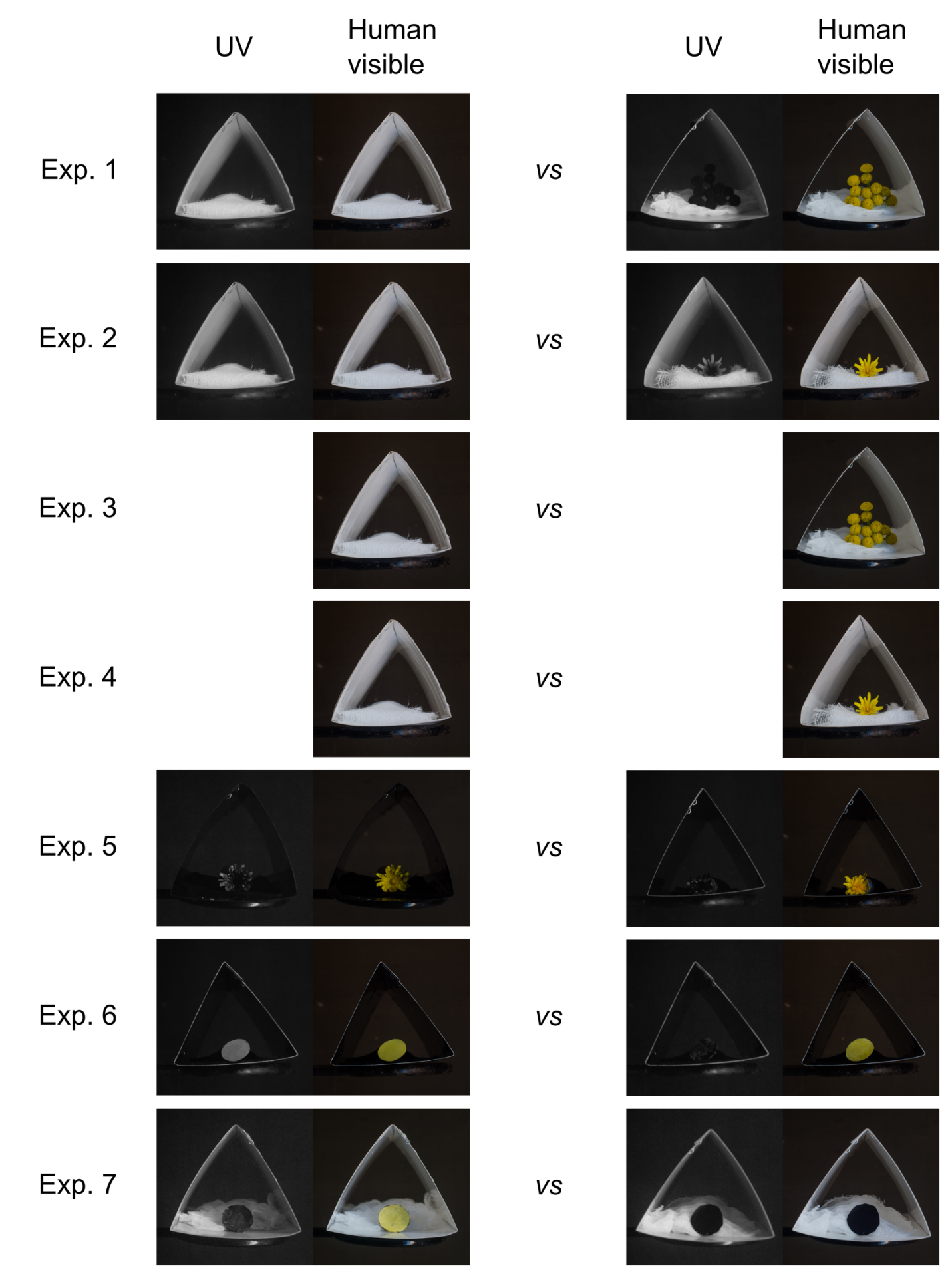

Supplement: S2 Fig — (TIF) [file pone.0217484.s002.tif]
